# Development of an integrated high-voltage electron microscope–gas chromatograph–quadrupole mass spectrometer system for the *operando* analysis of catalytic gas reactions

**DOI:** 10.1093/jmicro/dfae010

**Published:** 2024-02-27

**Authors:** Longshu Tang, Tetsuo Higuchi, Shigeo Arai, Hiromochi Tanaka, Shunsuke Muto

**Affiliations:** Graduate School of Engineering, Nagoya University, Nagoya 464-8603, Japan; JEOL Ltd., 3-1-2 Musashino, Akishima, Tokyo 196-8558, Japan; High-Voltage Electron Microscope Laboratory, Institute of Materials and Systems for Sustainability, Nagoya University, Nagoya 464-8603, Japan; Advanced Material Engineering Division, Toyota Motor Corporation, Susono 410-1193, Japan; Graduate School of Engineering, Nagoya University, Nagoya 464-8603, Japan; High-Voltage Electron Microscope Laboratory, Institute of Materials and Systems for Sustainability, Nagoya University, Nagoya 464-8603, Japan; Advanced Measurement Technology Center, Institute of Materials and Systems for Sustainability, Nagoya University, Nagoya 464-8603, Japan

**Keywords:** high-voltage electron microscopy, gas chromatograph, mass spectrometer, operando measurement, environmental cell, catalytic reaction

## Abstract

This paper describes the development of a gas chromatography–quadrupole mass spectrometry system attached to a differential-pumping-type environmental cell of the reaction science high-voltage electron microscopy instrument at Nagoya University to distinguish unambiguously between different gas species with the same mass-to-charge ratio. Several model experiments were used to verify the efficacy of the newly proposed system, confirming its ability to analyse the atomic-level structural changes during heterogeneous catalysts and the associated gas-reaction kinetics simultaneously, providing new insights into *operando* measurements in the field of environmental transmission electron microscopy.

**Graphical Abstract** 
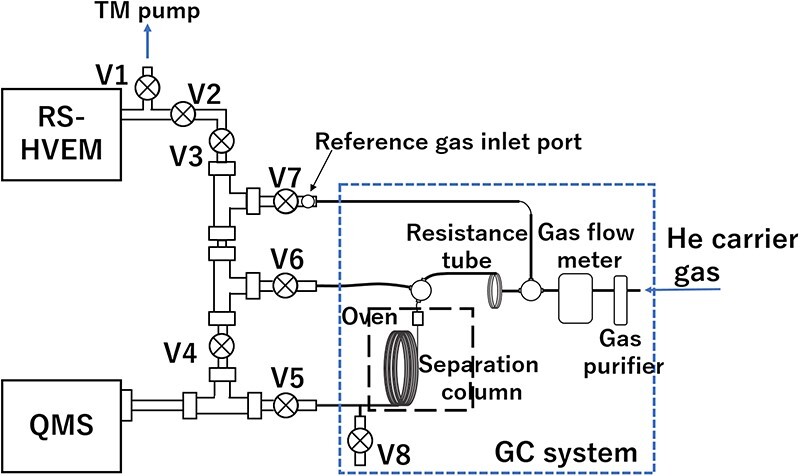

TEM, which has a significantly higher spatial resolution than most other observation/measurement methods, is widely used in natural science research. However, traditional TEM systems require the high-energy electron beam illumination of the target sample under vacuum; owing to this intrinsic limitation, and these TEM systems can only be used for the analysis of metals and inorganic thin films (i.e. ‘charred dried sardines’) [1]. With the technological development of TEM instrumentation, this limitation has been overcome by using specially designed specimen holders containing high-pressure reactive gases or liquids confined between thin electron-transparent membranes that enable structural analysis during chemical reactions [2,3] and micro-electro-mechanical systems (MEMS) that enable biasing and/or the application of mechanical force onto the sample [3–5]. In addition, a dedicated environmental TEM (e-TEM) instrument with a differential pumping system has been developed for efficient TEM analysis. In these systems, to confine the gas flow within the environmental cell (e-cell) and minimize gas leakage to the TEM column through the electron path out of the chamber, a reactive gas is intentionally introduced into the specimen chamber while evacuating the e-cell by using a powerful pumping system [6–9]. TEM data collected under such dynamically changing conditions are labelled *in-situ* TEM observations. *Operando* measurements, which represent an extension of this concept, indicate the structural changes and spectroscopic information of samples under a variety of different operating conditions (such as with catalysts and electric devices) [10].

The spatial resolution of TEM is proportional to ${({C_{\mathrm{S}}}{\lambda ^3})^{1/4}}$, where *C*_S_ is the spherical aberration coefficient and *λ* is the wavelength of incident electrons [11]; therefore, a higher accelerating voltage indicates a smaller electron wavelength and higher spatial resolution. Consequently, in the initial stages of TEM research, several scholars competitively attempted to increase the accelerating voltage of the incident electrons in TEM. Since 1964, the Nagoya University has developed high-voltage TEM instruments with accelerating voltages higher than 500 kV in collaboration with Japanese TEM manufacturers, excluding the 300 kV TEM system manufactured in 1954 [12], confirming the benefits of a high electron-penetration power at high accelerating voltages.

In the 1990s, the practical implementation of aberration-correction technology in TEM caused revolutionary developments in TEM instrumentation [13,14]. Instead of reducing the electron wavelength to improve the spatial resolution, the *Cs* of the objective lens was minimized without reducing the objective-piece gap smaller; rather, this design strategy resulted in TEM systems with the same spatial resolution as conventional TEM instruments but with a wider pole-piece gap, enabling the incorporation of thick specimen holders with multiple integrated MEMS. These technological advances rendered HVEM (with an intrinsic cost–performance imbalance) obsolete. Currently, HVEM instrumentation is not used in most countries (except Korea and Japan).

The fifth-generation high-voltage scanning TEM system, JEM1000K RS, built at Nagoya University in 2008–9 (named the reaction science HVEM system, i.e. RS–HVEM) [15] exhibits the advantageous features of several previous generations of HVEM instruments. It combines the *in-situ* experimentation of third-generation HVEM systems [16], including the wide pole-piece gap in the objective lens, with the beam-scanning system and electron energy-loss spectrometer of fourth-generation HVEM systems [17]. Moreover, it contains a retractable e-cell equipped with a differential pumping system, which enables atomic-resolution TEM observations, even in a gaseous atmosphere with a high total pressure of 10 000 Pa [15].

Besides investigating gas reactions [18–20], the current version of RS–HVEM is used in a wide range of research fields including materials science [21–25] and biology; the high electron-penetration power of the beam-scanning mode of electron tomography (i.e. the scanning TEM mode) is particularly useful for three-dimensionally observing samples that are a few microns thick [26–28]. Notably, most latest TEM systems are based on the principle of high resolutions at high magnifications, and the back focal plane of the objective lens is located just below (<1 mm) the sample in the pole-piece gap; thus, the objective aperture cannot be inserted exactly at the back focal plane, which makes the design of these TEM systems challenging. In principle, it is not possible to set both the Bragg diffraction spots and the objective aperture concurrently in focus in the diffraction mode. Moreover, a hollow shadow corresponding to the objective lens aperture appears in the image at low magnifications in the bright- and dark-field imaging modes. Therefore, HVEM enables the construction of classical and authentic TEM systems that use classical diffraction-contrast imaging to observe thick samples.

TEM systems equipped with e-cells are currently unsurpassed in their ability to observe dynamically the structural changes associated with catalytic gas reactions at the atomic scale under various conditions such as heating, cooling and electric-field biasing. However, the RS–HVEM analysis of actual gas-reaction experiments yields irreproducible results. Repeated trials under identical experimental conditions (such as temperature and gas pressure) generate different data, possibly because it is difficult to recreate identical conditions during different trials of the same experiment owing to variations in the gaseous environment of the TEM chamber with the experimental history of the system. The gas-chamber atmosphere typically includes a significant amount of ambient gas (N_2_ and O_2_) or residual gases from previous experiments. Hence, to ensure precise analysis, a gas-chamber purging procedure should be conducted before each experiment. Moreover, to account for variations in the e-cell gas environment in an experimental session (or in multiple sessions of the same experiment under similar conditions), it is vital to monitor and control the type and amount of gas species in the e-cell during experimentation. Consequently, the latest RS–HVEM instruments are equipped with QMSs [29].

In an electron-impact-ionization-type MS, the gas molecules under examination initially undergo electron impact, which causes their ionization by the loss of one or more electrons and fragmentation into smaller charged fragments. Subsequently, these positively charged ions are directed into the separation section by a repair voltage. The Coulombic forces from the separation electric field cause the separation and selective filtration of ionized molecules with different masses. Ultimately, these molecules are transformed into electrical signals that are amplified for monitoring and analysis. This enables the detection of trace gases at the parts per million or parts per billion level. However, as indicated by the separation principle of MS, it is challenging to separate target gases and fragments with identical mass-to-charge ratios (*m/z* values), such as N_2_^+^ and CO^+^ (*m*/*z* 28), CO_2_^+^ and N_2_O^+^ (*m*/*z* 44) and CH_4_^+^ and O^+^ (*m*/*z* 16), that are involved in several important catalytic-reaction systems, by using this method.

Notably, RS–HVEM–QMS systems have been utilized to investigate the purification of automotive-exhaust gases by ceramic-supported noble-metal-particle catalysts by analysing the structural changes in the metal-particle surface morphology owing to catalytic reactions and the time-dependent mass spectra of the emitted/consumed gas species (to elucidate the reaction kinetics of the system) [30]. This study aimed to develop an *operando* TEM-measurement system by incorporating GC into an RS–HVEM–QMS system, thereby extending the ability of the RS–HVEM–QMS system to distinguish between gas species with the same *m/z* value.

In this study, an Agilent 8890 GC system was attached to an HVEM–MS system; a block diagram of the resultant RS–HVEM–GC–QMS system is shown in Fig. 1; a detailed instrumental diagram of the HVEM–QMS system including an e-cell can be found in [29]. During analysis, the sample gas was transported through the separation column by high-purity He (99.99995%) (an inert carrier gas) to ensure minimal baseline noise. In addition, an HP2 heated He purifier (VICI Valco Instruments Co. Inc) was used to reduce the concentration of impurities, such as H_2_O, N_2_ and O_2_, to parts per billion levels, enabling trace-gas measurements. To ensure optimal separation by regulating the carrier-gas flow rate, a flow-rate control system comprising a mass-flow controller (SEC-E40MK3, HORIBA STEC Co. Ltd) that functioned as a gas operation unit was set up downstream of the gas purifier along with an associated control unit (PE-D20, HORIBA STEC, Co. Ltd) for the interactive control of the mass-flow controller.

**Fig. 1.**
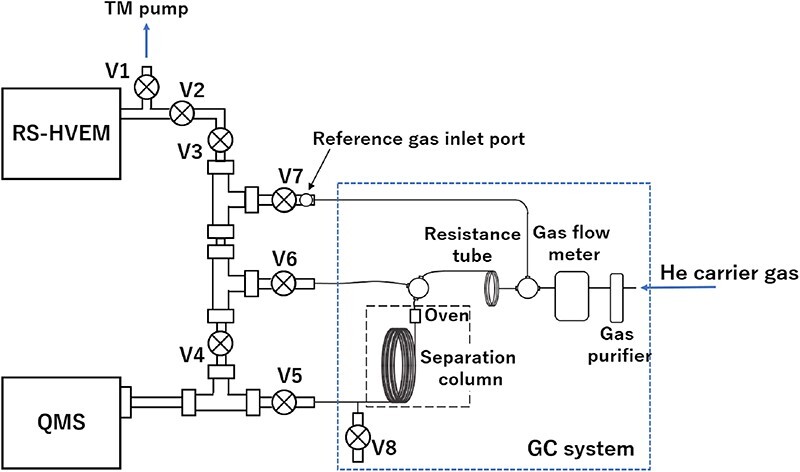
Block diagram of the newly proposed RS–HVEM–GC–QMS system. Abbreviation: RS–HVEM–GC–QMS, Reaction science–high-voltage electron microscopy–gas chromatography–quadrupole mass spectroscopy.

During GC testing, the tuned carrier gas flowed through the sample room and transported the analyte into the GC column. The components of a mixture of gas species can be separated by passage through a packed column owing to the unique interaction strength of each species with the column filling, which causes each species to pass through the column at a specific rate and generate a unique MS peak at a specific retention time.

A complete GC–MS test can be divided into four stages: the continuous MS mode, sampling, testing and the cessation of testing. Here, in the continuous MS mode, Valve #1 for the direct evacuation of the e-cell was closed; subsequently, Valve #2 was opened, Valves #3 and #4 were opened and Valves #5–7 were closed to guide the gas species in the e-cell to the QMS system through the liner tube. The carrier gas was released from exhaust Valve #8 after passing through the resistance tube and the GC system at a flow rate of 12 cc/min. During this stage, the gas components discharged from the e-cell were continuously monitored by the QMS system.

Sampling the e-cell gas comprised the first step of switching to the GC mode. Valves #4 and #3 were successively closed within 1 s to trap securely and sample the gas flowing through the linear tube between the e-cell and the QMS system. The prior closure of Valve #4 prevented the evacuation of the sample gas by the pump connected to the QMS system. Notably, the consecutive closure of Valve #3 is crucial as it avoids the excess introduction of gases from the e-cell, ensuring the accuracy of quantitative measurements. Subsequently, exhaust Valve #8 was closed and Valve #5 was opened to direct the carrier gas towards the QMS system. At this stage of analysis, the gas pressure within the sampling section was significantly lower (similar in magnitude to that in the e-cell) than the external pressure, because the carrier gas in the pipeline was previously at atmospheric pressure. To prevent an abrupt carrier-gas backflow towards the low-pressure region of the system, which could lead to peak distortion during valve opening for switching to the GC mode, the internal and external pressures were equalized by temporarily opening Valve #7 for ∼5 s. The complete sampling procedure was concluded with an equilibration time of 10 min, which stabilized the carrier-gas flow and eliminated any background-gas interference. The newly proposed RS–HVEM–GC–QMS system is shown in Fig. 2.

**Fig. 2.**
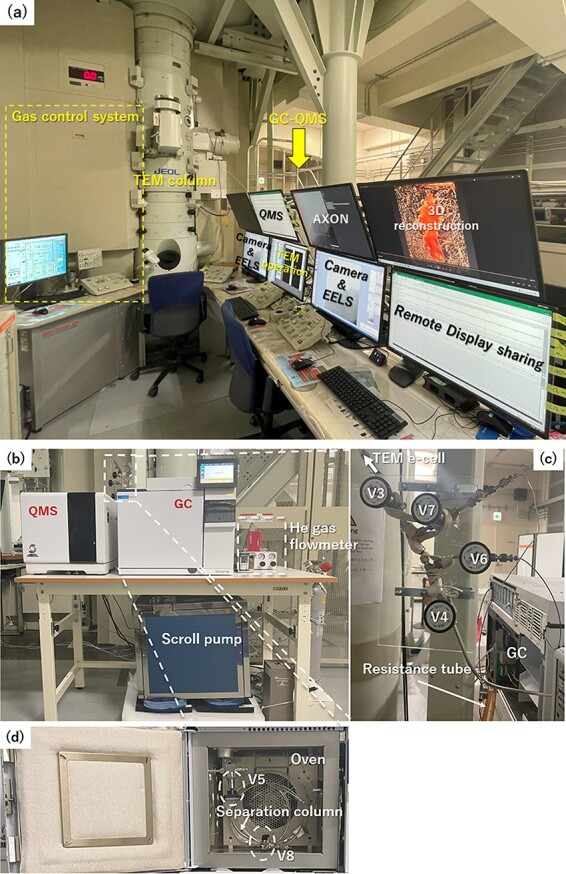
Photographs of the newly proposed RS–HVEM–GC–QMS system. (a) Front view of the RS–HVEM system; the GC–QMS system is attached to its rear (to the left). (b) External appearance of the GC–QMS system. (c) Valve system at the rear of the GC system. (d) Interiors of the GC oven with an open front cover. Abbreviation: RS–HVEM–GC–QMS, reaction science–high-voltage electron microscopy–gas chromatography–quadrupole mass spectroscopy.

Valves #6 and #7 were opened simultaneously to initiate GC testing. The prepared sample gas was transferred into the GC and separated by a column. The GC-testing temperature program was initiated concurrently with the opening of Valves #6 and #7, and MS spectra were recorded from this point (considered to be the zero point).

To terminate and switch back to the regular MS mode, Valve #5 was closed and exhaust Valve #8 was opened successively. This sequence prevented immediate air ingress through exhaust Valve #8, protecting the equipment from potential damage. Subsequently, Valves #6 and #7 were closed to redirect the carrier gas through the resistance tube and release it into the atmosphere. Finally, to reintegrate the QMS into the e-cell pipeline safely, Valves #3 and #4 were opened gradually.

The separation of CO^+^ from N_2_^+^ (with *m*/*z* 28) and CO_2_^+^ from N_2_O^+^ (with *m*/*z* 44) is an important aspect of research on the gas-reaction mechanisms of automotive-emission purification by heterogeneous catalysts (such as ceramic-supported metallic nanoparticles). After experimentation with several types of chromatographic columns, a carbon-packed column was identified as optimal for the separation of the aforementioned gas species. Owing to the high sensitivity of QMS systems to gas species at extremely low concentrations (ppm or ppb levels), signals corresponding to N_2_ and CO_2_ are always detected in the atmospheric background, which sometimes smear out the signals of the counterpart gas species (i.e. CO and N_2_O, respectively). Thus, for a comprehensive understanding of the catalytic reaction mechanisms of automotive-emission purification under various conditions, it is crucial to monitor unambiguously CO as an automotive-emission gas and the N_2_O generated as a result of the imperfect reduction of nitrogen oxides. The experimental conditions and instrumental parameters used in this study are listed in Table 1.

**Table 1.** Experimental conditions and instrumental parameters used in the present study

		CNT combustion		
	Reference gas (CO, N_2_, CO_2_, CH_4_, C_2_H_6_)	with Pd	without Pd (Blank)	Rh/ZrO_2_ model catalyst
EM voltage for GC mode (V)	1300	1250		1250
Ionization current (μA)		20		
Ionizing energy (eV)		70		
MS scan rate (frames/s)		2		
GC carrier-gas flow rate (ml min^–1^)		12		
Temperature history of GC oven	Step 1: 30℃: 10 min Step 2: 30℃ → 80℃ (5℃ min^–1^) Step 3: 80℃ (constant)			
Introduced gas pressure	N/A	O_2_: 5 Pa		3% NO/3% CO/94% Ne: 45 Pa
Sample heating temperature (℃)	∼300	800		700
Base pressure of GC–QMS	1.9 × 10^−4^ Pa (at 30 Pa gas atmosphere in e-cell)			
Gas sampling capacity for GC operation		3.4 cc		

Generally, the main challenge associated with S/TEM–QMS systems is to detect concurrently the reactants generated in the e-cell (without a retention time) while observing the corresponding structural changes associated with the reaction using TEM images. Several systems, such as residual gas analysers with commercial atmospheric-condition TEM specimen holders [31], are unable to detect the reactants and structural changes simultaneously as it is challenging to transport the e-cell reactant gases to the QMS system through the thin-line connecting tube using the scroll pump attached to the QMS alone. To mitigate this problem, the distance between the TEM and QMS should be reduced, thereby decreasing the length of the connecting gas-line tubes, which hamper atomic-level observations owing to the mechanical vibrations of the residual gas analysers. Notably, in this study, due to the direct connection between e-cell and QMS, the analytes reach the QMS and generate signals within 1 s, thereby circumventing analytical challenges arising from temporal discrepancies. In addition, owing to the large weight of the RS–HVEM system (∼50 tons), mechanical vibrations from the QMS pumping system influenced the atomic-resolution of TEM observations negligibly. This is an unexpected advantage of large-scale TEM instruments.

As a feasibility test, we examined whether the constructed GC–QMS system correctly detects N_2_ and CO separately and measured the approximate retention time of each. A commercially available standard gas mixture (0.1 cc) comprising CO, CH_4_ and other gas species (Table 1) was injected into the inlet port of the GC–QMS system attached to Valve #7. N_2_ should always be used as the background gas. The GC spectra for *m*/*z* 28 (CO^+^ and N_2_^+^), 12 (C^+^ fragment from CO) and 14 (N^+^ fragment from N_2_) are shown in Fig. 3. The *m*/*z* 28 spectrum contains two peaks at 5.4 and 7.1 min; a comparison of the *m*/*z* 12 and 14 spectra confirms that the peaks in the *m*/*z* 28 spectrum originate from N_2_^+^ and CO^+^, respectively. The present retention times for N_2_^+^ and CO^+^ of *m*/*z* 28 may deviate from those for the actual HVEM–GC–QMS system, because the valve operation sequence and the associated flow gas pressure may change. The identification of the same *m*/*z* ions should rely on the common retention time for conceivable fragment ions.

**Fig. 3.**
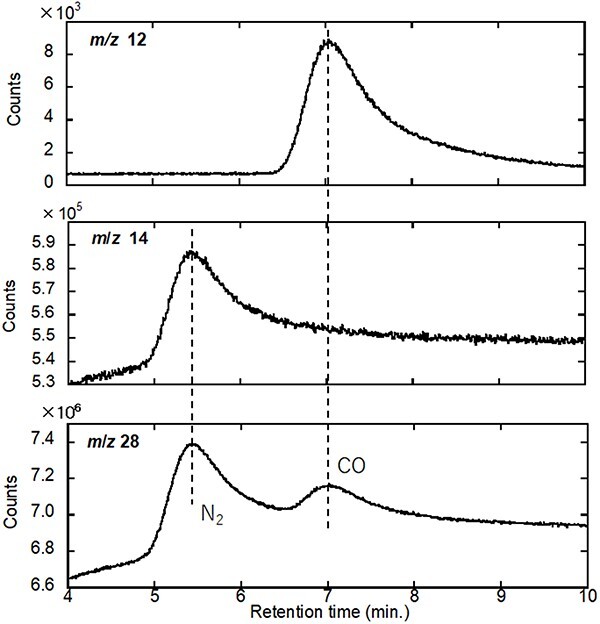
GC–QMS charts of *m*/*z* 12, 14 and 28 as a function of the retention time for gas analysis (see Table 1 for the gas-species composition of the analysed mixture). Abbreviation: GC–QMS, gas chromatography–quadrupole mass spectroscopy.

The sample used for the feasibility study of HVEM–QMS systems was employed to investigate the ability of the newly proposed TEM–GC–QMS system to separate CO and N_2_ (always present as a background signal) derived from the catalyst-assisted combustion of mixed carbon samples comprising carbon nanotubes (CNTs) and carbon black in an e-cell [29]. During analysis, a mixture of CNTs, carbon black powder and Pd nanoparticles dispersed in ethanol was mounted onto the W filament of a Kamino-type specimen-heating holder [32]. To induce imperfect C combustion for CO generation, the O-gas pressure in the e-cell was set to 5 Pa, which is one order of magnitude lower than the O-gas pressure used in the previously published CNT-combustion experiments [29], and the sample was heated to 800°C. After confirming the drastic consumption of CNTs by e-TEM, the aforementioned sampling procedure was applied for GC testing. The GC spectrum for *m*/*z* 12 is shown in Fig. 4a; for comparison, the GC spectrum recorded on repeating the same experimental procedure at room temperature is provided in Fig. 4b.

**Fig. 4.**
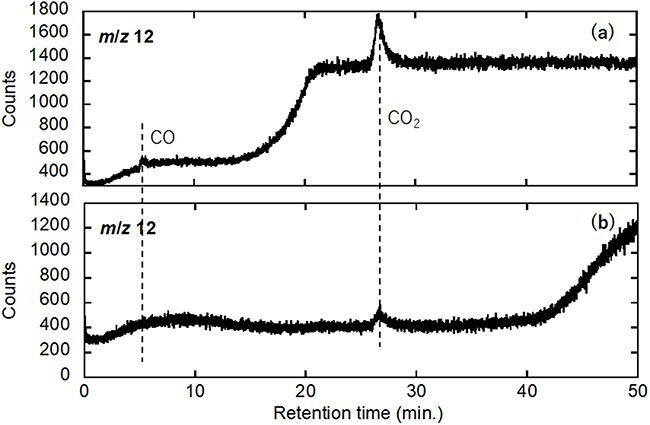
GC–QMS charts of *m/z* 12 as a function of the retention time for (a) Pd catalytic-particle-assisted CNT combustion in an oxygen atmosphere at 800℃ and (b) for a blank experiment (without Pd particles) at 800℃. Abbreviation: GC–QMS, gas chromatography–quadrupole mass spectroscopy.

The *m*/*z* 12 spectrum contains two successive peaks of the C^+^ fragment at the retention times of CO and CO_2_ (see Fig. 3 for CO_2_). The absence and presence of a C^+^ peak from CO and CO_2_, respectively, owing to the residual background gas in the room temperature experiment confirmed the assignment of the two successive peaks in the *m/z* 12 spectrum to CO and CO_2_. It should be noted that the retention time for the CO^+^ peak was approximately 5 min, which is 2 min shorter than that of the reference gas data for the reason mentioned earlier.

Finally, the ability of the newly proposed system to detect N_2_O and distinguish it from CO_2_ in the *m*/*z* 44 spectrum during NO conversion with a ZrO_2_-supported Rh-nanoparticle catalyst was tested using a previously published protocol [30]. Unlike the previous study, which used 3 mol% of NO/Ne gas for analysis, a gaseous mixture of NO and CO diluted with Ne (3 mol% NO/3 mol% CO/94 mol% Ne) was employed in this study. The ion-monitoring spectra selected by QMS for *m*/*z* 30 and 44 as functions of the temperature-ramping time are shown in Fig. 5a; these spectra confirm that the model catalyst system is active. Notably, the spectrum of *m*/*z* 30 can be attributed to NO^+^ (derived from the source gas and the produced N_2_O owing to imperfect reduction). The reactant gas was sampled at 700°C for GC analysis. The representative TEM image of an Rh particle (of the catalyst) at 700°C is shown in Fig. 5b; unlike the effects of NO injection alone, this Rh-particle surface is negligibly oxidized.

**Fig. 5.**
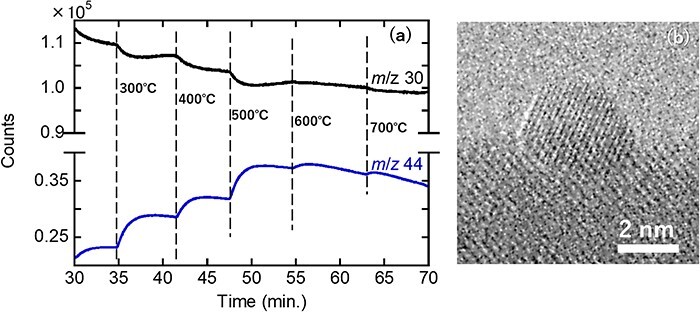
(a) Selected ion-monitoring QMS charts for NO–CO conversion experiments by a ZrO_2_-supported Rh-nanoparticle model catalyst. (b) High-resolution TEM image of an Rh particle of the catalyst in a CO–NO/Ne atmosphere (under a total pressure of 45 Pa) at 700℃. Abbreviation: QMS, quadrupole mass spectroscopy.

The GC spectra for *m*/*z* 44, 12 and 30 are shown in Fig. 6. The *m*/*z* 44 spectrum contains a very large peak (truncated) and a small peak. The large peak was attributed to CO_2_^+^ owing to its retention time; a similar peak with the same retention time was observed in the *m*/*z* 12 spectrum corresponding to the C^+^ fragment, confirming the validity of the previous assignment. The *m*/*z* 30 spectrum can be used to detect the presence of N_2_O unambiguously (among N, N_2_ and NO in the system), because NO^+^ is only derived from NO (the source gas) and N_2_O in this experiment. Figure 6 indicates two peaks in the *m*/*z* 30 spectrum; the first broad peak was attributed to the NO source gas owing to its relative intensity. Thus, the second peak at 13.5 min in the *m*/*z* 30 spectrum was attributed to the NO^+^ fragment from N_2_O that generated the smaller peak in the *m*/*z* 44 spectrum. In addition, the *m*/*z* 12 peak is observed at a retention time of ∼5 min; this peak should be derived from CO, one of the source gas species, consistent with that observed in Fig. 4.

**Fig. 6.**
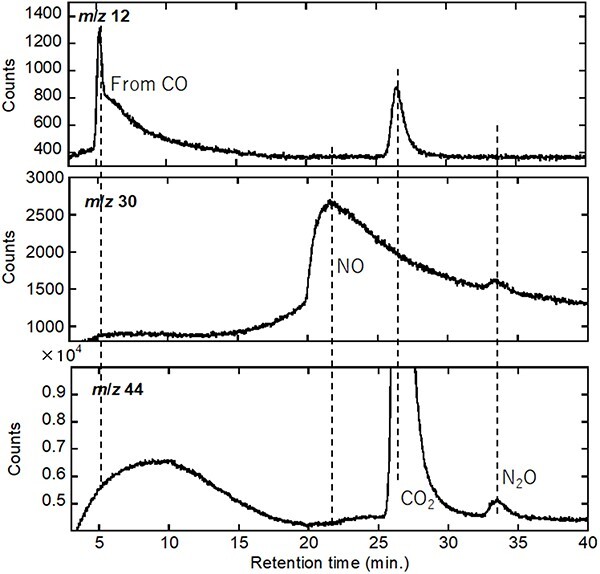
GC–QMS charts of *m*/*z* 12, 30 and 44 as a function of the retention time for NO–CO conversion testing by a ZrO_2_-supported Rh-nanoparticle model catalyst at 700℃. Abbreviation: GC–QMS, gas chromatography–quadrupole mass spectroscopy.

Despite the ability of the newly proposed TEM–GC–QMS system to distinguish between N_2_O and CO_2_ in the *m/z* 44 spectrum and between CO and N_2_ in the *m/z* 28 spectrum, it exhibits several limitations. First, valve operation for gas sampling in the proposed GC system obstructs the direct gas line between the e-cell of the TEM and the QMS system, interrupting stable gas flow. Thus, two separate lines are required for the continuous operation of the QMS and GC–QMS modes, necessitating the incorporation of two QMS systems into the testing setup. Moreover, the quantification of the reactant-gas components detected by QMS and GC–QMS is challenging owing to the distinct ionization yield of each gas species. In principle, quantification is feasible by using a standard gas with a known composition as the source gas. However, even with a known-composition source gas, it is difficult to quantify the gas species in the system, significant amounts of which are present in the ambient atmosphere (such as N_2_, O_2_, CO_2_ and Ar). Nevertheless, despite its limitations, the newly proposed GC system enables the concurrent observation of atomic-level structural changes during catalysis and the detection of dynamically varying amounts of reactant gases as a function of time, providing valuable insight into the kinetics and mechanisms of catalytic reactions.

## Data Availability

Not applicable.

## Abbreviations

S/TEM, Scanning/transmission electron microscopy; QMS, Quadrupole mass spectroscopy; GC, Gas chromatography; HVEM, High-voltage electron microscopy; RS–HVEM, Reaction science HVEM.
